# Development of a Parent Wireless Assistive Interface for Myoelectric Prosthetic Hands for Children

**DOI:** 10.3389/fnbot.2018.00048

**Published:** 2018-08-02

**Authors:** Yutaro Hiyoshi, Yuta Murai, Yoshiko Yabuki, Kenichi Takahana, Soichiro Morishita, Yinlai Jiang, Shunta Togo, Shinichiro Takayama, Hiroshi Yokoi

**Affiliations:** ^1^Department of Mechanical and Intelligent System Engineering, Graduate School of Informatics and Engineering, The University of Electro-Communications, Tokyo, Japan; ^2^Beijing Innovation Center for Intelligent Robots and Systems, Beijing, China; ^3^Brain Science Inspired Life Support Research Center, The University of Electro-Communications, Tokyo, Japan; ^4^National Center for Child Health and Development, Tokyo, Japan

**Keywords:** myoelectric prosthetic hand, EMG, human-machine interface, children, artificial neural network, threshold

## Abstract

In this study, a one-degree-of-freedom myoelectric prosthesis system was proposed using a Parent Wireless Assistive Interface (PWAI) that allowed an external assistant (e. g., the parent of the user) to immediately adjust the parameters of the prosthetic hand controller. In the PWAI, the myoelectric potential of use of the upper limb was plotted on an external terminal in real time. Simultaneously, the assistant adjusted the parameters of the prosthetic hand control device and manually manipulated the prosthetic hand. With these functions, children that have difficulty verbally communicating could obtain properly adjusted prosthetic hands. In addition, non-experts could easily adjust and manually manipulate the prosthesis; therefore, training for the prosthetic hands could be performed at home. Two types of hand motion discrimination methods were constructed in this study of the myoelectric control system: (1) a threshold control based on the myoelectric potential amplitude information and (2) a pattern recognition of the frequency domain features. In an evaluation test of the prosthesis threshold control system, child subjects achieved discrimination rates as high as 89%, compared with 96% achieved by adult subjects. Furthermore, the high discrimination rate was maintained by sequentially updating the threshold value. In addition, a discrimination rate of 82% on average was obtained by recognizing three motions using the pattern recognition method.

## Introduction

The myoelectric prosthetic hand is a robotic device controlled by the myoelectric potential of the user, and it functions as the hand of an upper-limb deficient person. A direct control includes threshold control and proportional control using the myoelectric potential of specific locations (e.g., the extensor muscle group of the forearm, or the flexor muscle group). This is a typical prosthetic control method (Otto Bock Healthcare Products GmbH, 2013a; Powell and Thakor, 2013). In recent years, the pattern recognition control method of using a prosthetic device has also been studied (Nishikawa et al., 1999; Zecca et al., 2002; Micera et al., 2010; You et al., 2010; Hasan et al., 2014; Jiang et al., 2014; Manea et al., 2015; Ke et al., 2016). The use of myoelectric prosthetic hands with multiple Degrees Of Freedom (DOF) is simpler in the pattern recognition method than in the direct control method. In direct and pattern recognition control methods, training by experts, such as occupational therapists, is important (Lake, 1997; Marcus et al., 2009; Powell and Thakor, 2013).

Myoelectric prosthetic hands are primarily intended for adults. They are manufactured and sold as commercial products (Otto Bock Healthcare Products GmbH, 2013b; RSL STEEPER, 2014) and myoelectric prosthetic hands for children are also sold by several companies (Fillauer, 2010; VASI, 2012; RSL STEEPER, 2014; Otto Bock Healthcare Products GmbH, 2016). For a congenital upper limb amputee, it is desirable to use and train myoelectric prosthetic hands at an early stage to reduce the future rejection rate (Routhier et al., 2001; Toda et al., 2015). Therefore, the development and improvement of the myoelectric prosthetic hand for children is important. Some studies have developed prosthetic hands for children (Redman et al., 2011; Zuniga et al., 2016; Mounika et al., 2017). However, the limitation of hand size and the difficulty in clinical testing make the study of prosthetic hands for children difficult. Child users also require training for the myoelectric prosthetic hands; however, in general, the length of time that a child can concentrate on training is less than his or her age plus 1 min (The Student Coalition for Action in Literacy Education, 2014). Therefore, it is difficult for children to concentrate during long-term training. There is also a limitation in verbal communication with children, and it is difficult to obtain efficient training in a short time. Owing to the above and other factors, the mean rejection rate of electric prostheses was 35% in pediatric populations (Biddiss and Chau, 2007).

One training method used a parental switch to train a child on a myoelectric prosthetic hand that performed direct control (Muzumdar, 2012; VASI, 2012). By using a switch, an external assistant intervened in the movement of the myoelectric prosthetic hands; therefore, if the child could not understand the verbal explanation, the child could notice the myoelectric prosthetic hand movement. In the pattern recognition method, the control method is fundamentally different from the direct control method; therefore, it is difficult to control the myoelectric prosthetic hand without the child's understanding. Training data, such as measured myoelectricity from the user based on operation intention, are required. However, there is no myoelectric prosthetic hand system that uses external assistance in pattern recognition, such as a parental switch in direct control.

Therefore, in this study, a system with control parameters that could be adjusted by external assistance is proposed. This system can be implemented with the direct and pattern recognition control methods. Using this system, a myoelectric prosthetic hand system that could be used by a child was constructed. The requirements of the proposed myoelectric prosthetic hand system are as follows.

① The hand system must be usable by children who cannot understand verbal communications.
② The hand system should contain functions of the direct control and pattern recognition methods.
③ The parental switch should allow an external assistant to control the myoelectric prosthetic hand at any time.
④ The hand system should be easy to use, and it should be usable by non-specialists (e.g., the parents of the users).

To satisfy the above requirements, a system that allowed the external assistant to view the myoelectric potential of the user in real time and to change the control parameters and prosthetic hand action was developed. The proposed system was referred to as the Parent Wireless Assistive Interface (PWAI), in which the myoelectric potential was observed and the parameters of the controller could be adjusted at any time while the user was wearing the myoelectric prosthetic hand in direct control. By adjusting the threshold value according to the myoelectric status of the user, the prosthetic hand could be used without involving the user in the adjustment. In addition, in pattern recognition, real-time monitoring of the myoelectric patterns and selection of the training data by the assistants were possible. Through this assistance, the myoelectric prosthetic hand could be used without the user being conscious of the training data collection step. For ease of use, an Android terminal and a Windows personal computer with a popular mobile interface were used.

## System configuration

**Figure 1:**
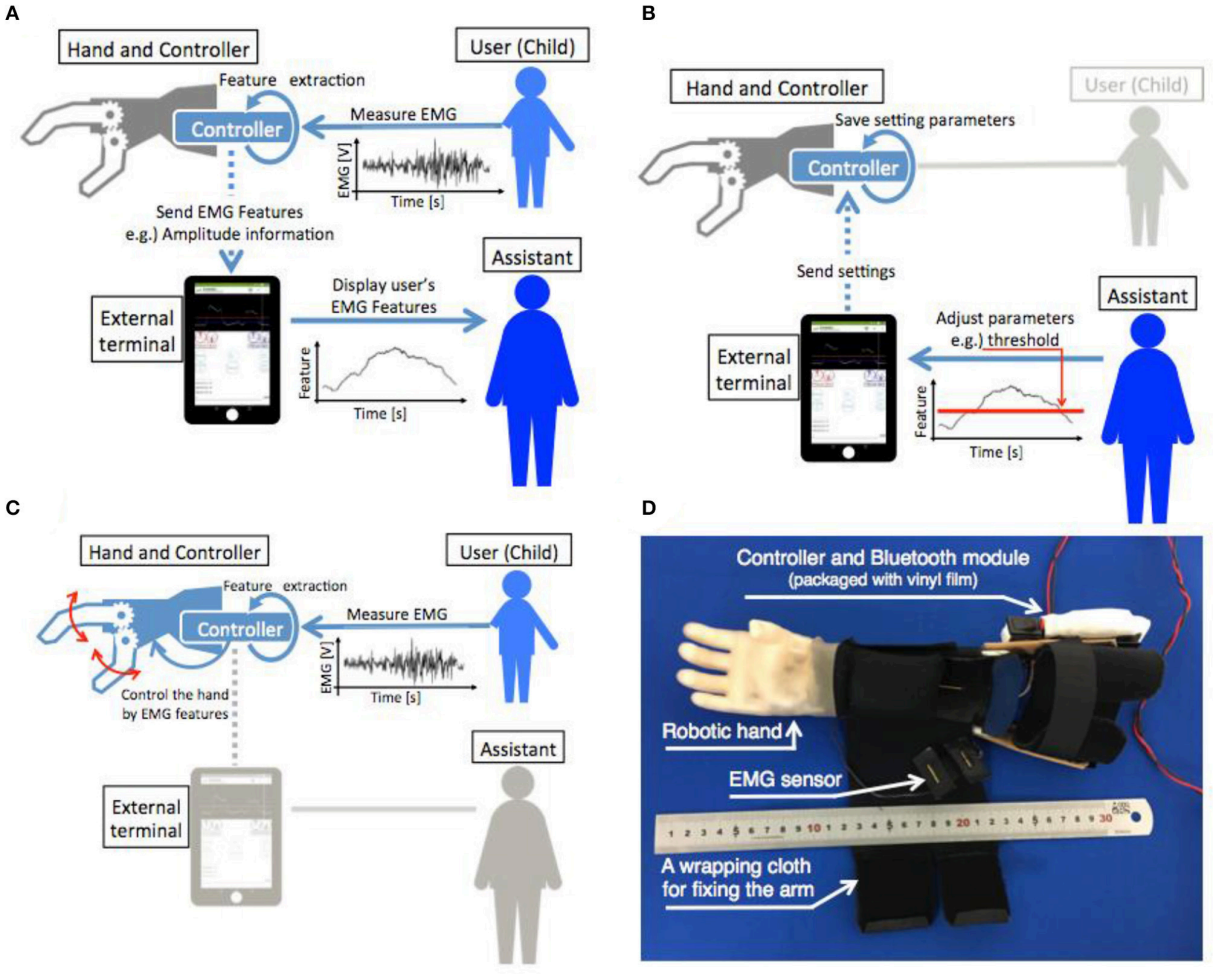
Schematic of the information flow between the components. **(A)** Shows the flow of information from the EMG measurement to the myoelectric characteristic feature presentation for the assistant. **(B)** Shows the parameter adjustment of the prosthesis controller by an assistant via an external terminal. **(C)** Shows the control of the prosthetic hand by user EMG. **(D)** Shows the developed myoelectric prosthetic hand for children after assembly.

The child myoelectric prosthetic hand system was divided into four components: the myoelectric sensor, prosthesis controller, one-DOF hand, and external terminal. Figures 1A–C show the transformation of the information between each component. In addition, Figure 1D shows a photograph of the child myoelectric prosthetic hand.

### EMG sensor

**Figure 2:**
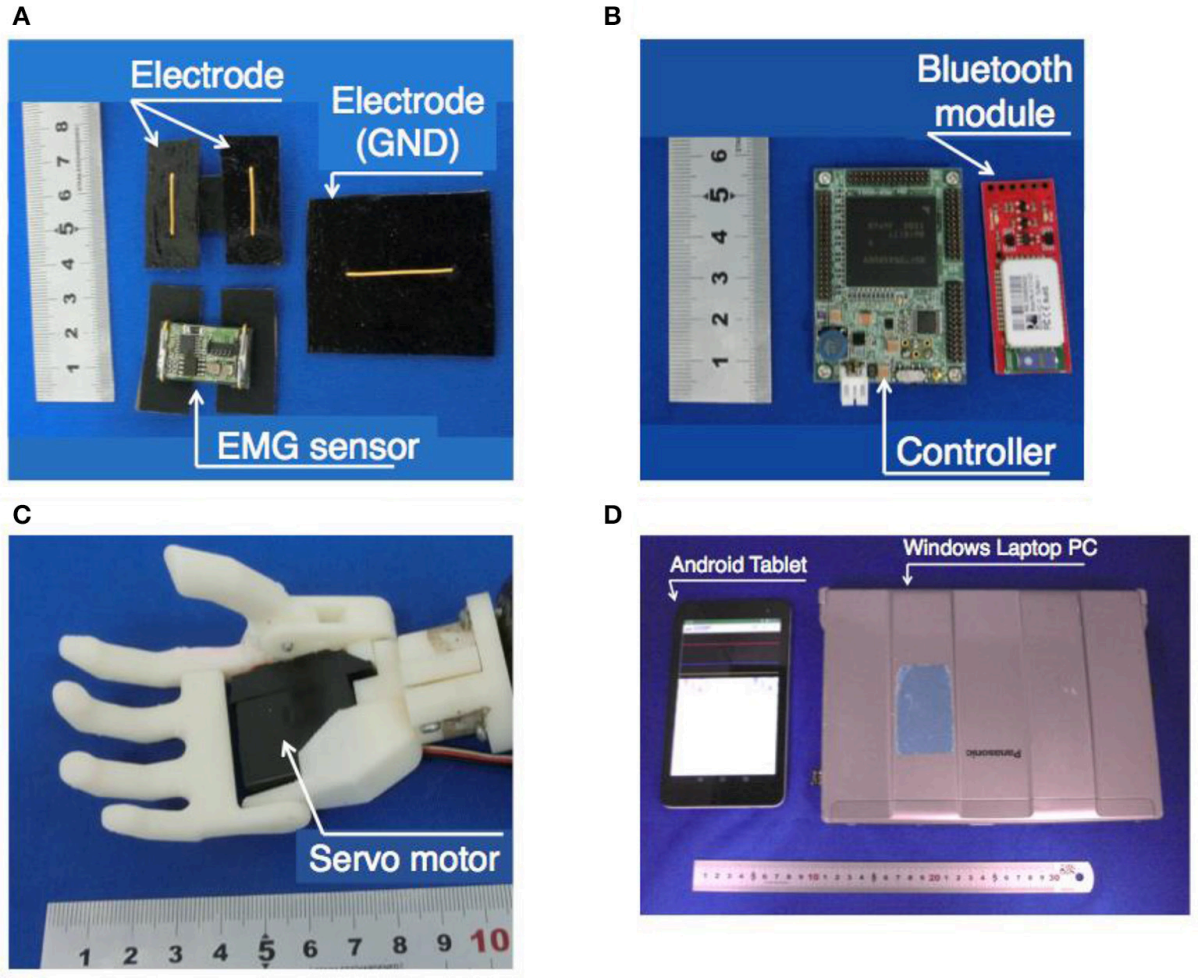
Appearance of each element of the myoelectric prosthetic hand system for a child. **(A)** EMG sensors, **(B)** controller and Bluetooth module, **(C)** robotic hand prosthetics, and **(D)** external terminals.

Figure 2A shows the sensor including an electrode. This sensor was previously developed by our research group (Jiang et al., 2017). For the myoelectric sensor, a differential amplifier circuit with an integrated circuit (AD620, Analog Devices, Japan) was used. In this system, one myoelectric sensor and one body ground were used. The gain of the differential amplifier circuit was set to 100. Then, a filtering process was performed using a twin-T, 50 Hz notch filter, an active low-pass filter with a cutoff frequency of 100 Hz, and an active high-pass filter with a cutoff frequency of 10 Hz. The signal was amplified 474 times by the non-inverting amplifier circuit. Finally, the output was obtained. The signal was offset by +2.5 V with a range of 0–5 V. The electrode of the sensor had a gold-plated wire with a diameter of 1.0 mm and a conductive silicon resin mixed with 3% carbon.

### Hand controller

The controller andBluetooth module are shown in Figure 2B. The controller of the prosthetic hand was a SH-72544R micro-controller board (REK-0001, Kyoei Sangyo, Japan and SH-72544R, Renesas, Japan). The controller used analog to digital conversion to change the output of the analog value from the myoelectric sensor with a quantization of 12 bits and a sampling rate of 2 kHz, and performed the processing for the hand control. The details of the processing are described in section Proposed Method of the PWAI. The controller outputted the estimated hand motion from the myoelectric information and sent a control signal to the servomotor that was built into the hand. The feature-extracted myoelectric information was transmitted to the external terminal and was used for controlling the hand. The communication between the external terminal and the controller was performed at 115,200 bps via a Bluetooth module (Bluetooth Mate Silver WRL-12576, SparkFun, USA).

### Robotic hand

The robotic hand is shown in Figure 2C. Because a child prosthetic hand should be compact and lightweight, a skeleton of the hand was made with a 3D printer using ABS resin (Jiang et al., 2014; Jing et al., 2014; Curline-Wandl and Ali, 2016). One servo motor (GWSMICRO/2 BBMG/J, GWS, Taiwan) was incorporated into the palm, and the motor output shaft was connected to the four-finger root. The thumb was arranged to face the four fingers, and at the base of the thumb, it was connected to the four-finger rotational axis with a spur gear. The thumb was designed to move simultaneously with the movement of the four fingers. To create a natural appearance and to improve the gripping performance, a glove made of a styrene elastomer was attached (Yabuki et al., 2016).

### External terminal

For the external terminal, a Bluetooth communication function was required. In this study, an Android tablet (MediaPad T1 7.0, Huawei, China) and a Windows laptop PC (Let's Note CF-S9, Panasonic, Japan) were used. The terminals used in this research are shown in Figure 2D. The external terminal showed the myoelectric feature graph to the assistants in real time. The myoelectric characteristic level was transmitted from the prosthesis controller to the external terminal via Bluetooth. The assistant could also adjust the parameters of the prosthesis controller via the external terminal. The detailed contents of the graphs and parameter adjustment systems are described in section Proposed Method of the PWAI.

## Proposed method of the PWAI

The most commonly used myoelectric prosthetics hands for children perform threshold control using myoelectric potential amplitude information (Fillauer, 2010; VASI, 2012; RSL STEEPER, 2014; Otto Bock Healthcare Products GmbH, 2016). In the conventional method, the assistant initially measures the user's myoelectric potential and then adjusts the gain of the sensor circuit according to the magnitude of the myoelectric potential. By adjusting the sensor amplitude, the prosthetic hands can absorb individual differences in myoelectricity. However, measurement, adjustment, and fitting must be performed in order, and it is difficult to adjust each time the prosthetic hand is worn. Therefore, in this study, a system was developed that used the amplitude information of the EMG for prosthetic control and allowed the assistant to monitor on an external terminal and adjust the threshold at any time. In addition, although a majority of the myoelectric prosthetic hands for children have only one DOF, this system enables parameter adjustment in the pattern recognition control method for multi-DOF prosthetic systems. For adult multi-DOF EMG prosthetic hands, a method of pattern recognition is more effective than obtaining a combination of threshold value identifications from multiple myoelectric sensors (Amsuess et al., 2014). Therefore, the identification of hand motion by myoelectric potential pattern recognition has been studied extensively (Nishikawa et al., 1999; Zecca et al., 2002; Micera et al., 2010; You et al., 2010; Zhang et al., 2013; Hasan et al., 2014; Kasuya et al., 2015; Manea et al., 2015; Ke et al., 2016). In this study, we propose an external assistance method suitable for multi-DOF control using the pattern recognition technique. To generate the training data, the user was required to output the EMG information corresponding to the motion to be identified. Although this was an easy procedure for adult users, it is extremely difficult for child users. In the proposed method, the assistant selects the training data by using the external device. By using the training data selected by the assistant, it is possible to classify the intentions of multi-DOF motion even if the user was a child.

### Threshold control method

In the threshold control method, the amplitude information of the EMG measured from one sensor was used as the feature value *Y*_*t*_[V] at time*t*[*s*]. In this method, the threshold for identifying the muscle tension (higher threshold: *θ*_high_[V]) and the threshold for determining the muscle weakness (lower threshold: *θ*_low_[V]) were set. When the feature value *Y*_*t*_ was below *θ*_low_, the weakness was assigned to the opening motion of the prosthetic hand. In addition, when the feature value *Y*_*t*_ was higher than *θ*_high_ and the muscle tension was applied, the muscle contraction state was assigned to the grip operation. The section between the two thresholds was defined as the insensitive area, and the prosthetic hand did not move. During rest, grasp, and open motions, the motion identifiers were assigned as shown in Equation (1), and the hand motion was identified as shown in Equation (2)

(1) $$\begin{array}{c} {\text{M}}_{\text{ID}}=\{\begin{array}{cc} 0 & \text{stop}\\ 1 & \text{open}\\ 2 & \text{grasp} \end{array} \end{array}$$

(2) $$\begin{array}{c} \text{motion }(Y_{t})=\{\begin{array}{cc} 0 & \left(\theta_{\text{low}}\leq Y_{t}\leq \theta_{\text{high}}\right)\\ 1 & \left(Y_{t}<\theta_{\text{low}}\right)\\ 2 & \left(\theta_{\text{high}}<Y_{t}\right) \end{array} \end{array}$$

#### Myoelectric potential amplitude feature

The myoelectricity feature value was calculated with a cycle of 10 ms, and identification processing was performed using the latest value. For preprocessing the myoelectric potential, a second-order IIR high-pass filter with a cutoff frequency of 50 Hz was applied to reduce the noise caused by body movements.

To extract the amplitude information, the exponential moving average was obtained using the 256 full-wave rectified sampled data and the feature value of the previous step. The parameter *a* was the smoothing coefficient, and *b* was (1 − *a*). The parameter *X* was the sampling data after high-pass processing, and *Y* was the feature level. The parameter *S* was the intermediate variable. Then, a new feature quantity *Y*_*t*+1_ was obtained by Equation (3). In this study, a = 0.9999 and b = 0.0001.

(3) $$\begin{array}{l} S_{0}=Y_{t}\\ S_{i+1}=aS_{i}+b|X_{i}|\;\{i|0\leq i\leq 255\}\\ Y_{t+1}=S_{256} \end{array}$$

#### Graph drawn on the external terminal

**Figure 3:**
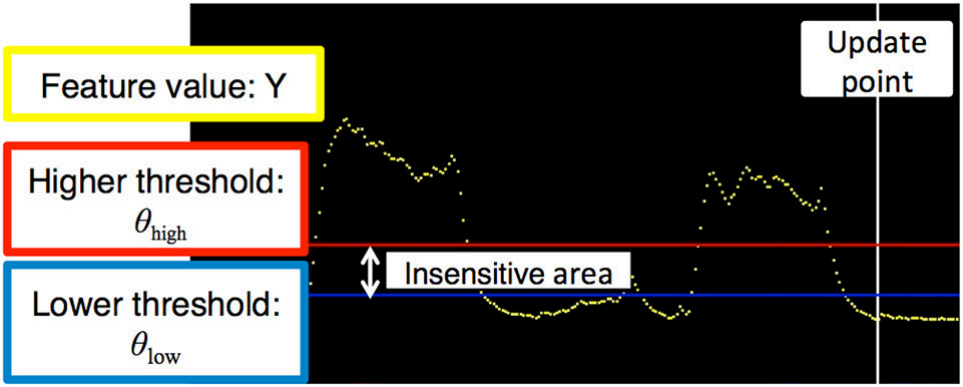
Graph of the threshold control method. The horizontal and vertical axes denote time and voltage, respectively. The yellow dot indicates the exponential moving average of the measured electromyogram. The red and blue horizontal lines are higher and lower thresholds, respectively. The vertical white line is the update point in the graph.

The myoelectric features were transmitted to the external terminals at 100 ms intervals and plotted as a graph with the vertical axis as the voltage (Figure 3). To reduce the weight of the communication data, the feature quantities were quantized in 256 steps in the range of 0–0.25 V when transmitted. The transmission data were transmitted in order, 5 bytes at a time. The first byte of data was the signal for the start of communication. The other 4 bytes of data were transmitted as 1 byte for the feature quantity, 1 byte for the higher threshold, 1 byte of the lower threshold, and 1 byte for the sum of the feature quantity and two threshold values. After the external terminal received the data, the oldest buffered feature quantity was overwritten with the newest feature quantity, and the newest feature quantity was plotted on the graph. When the value of the received threshold was different from the value stored in the external terminal, the new threshold value was updated and drawn on the graph. The red horizontal line indicated the higher threshold, and the blue horizontal line indicated the lower threshold. The yellow dot indicated the feature value, and it became a larger value as it approached the top of the screen. The white vertical line indicated the updated portion of the graph.

#### Operation of the external terminal

**Figure 4:**
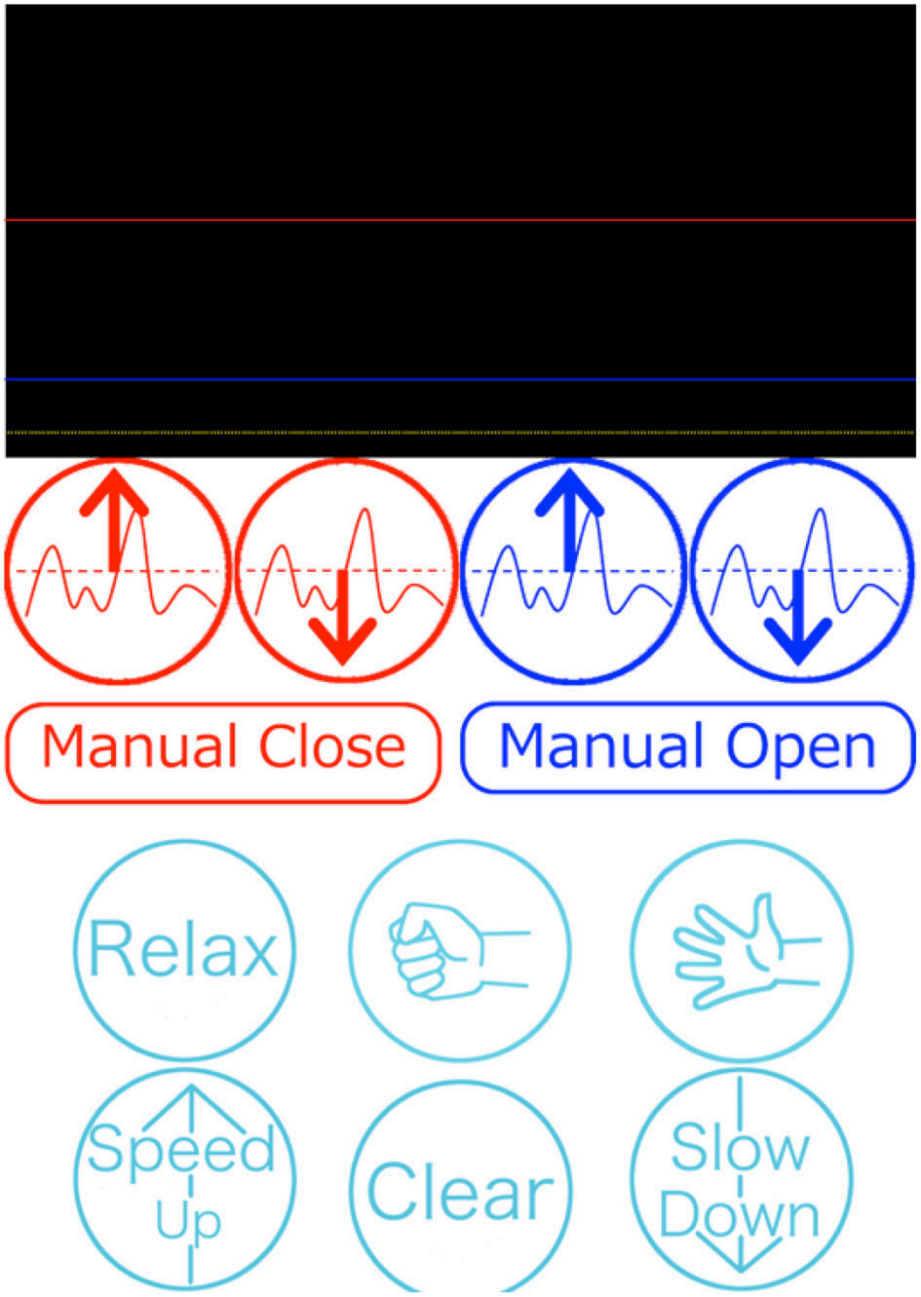
Presentation of the external terminal software program. The upper part shows information related to threshold control (Figure 3). The middle red and blue buttons are used to adjust the thresholds and to manually control hand motion. The bottom light blue buttons are used for the pattern recognition control method.

In the external terminal, the assistant could adjust the two threshold values and open and close the prosthetic hand with a manual operation. The external terminal software presentation is shown in Figure 4. The values of the threshold were stored in the prosthetic controller. When the prosthesis could not be moved as intended by the user, or when the motion of the prosthesis became unstable owing to a change in the skin condition, the assistant could re-set the threshold value θ = (*θ*_high_, *θ*_low_).

When the assistant adjusted the threshold value θ, the graphically drawn past threshold θ_old_ and the myoelectric feature values *Y* were used to determine whether to raise or lower the threshold value θ. This operation, performed by the assistant, was defined as Equation *O*(*θ*_old_, *Y*) in this study. The reconfigured threshold *θ* was overwritten on the prosthesis controller; see Equations (4–6)

(4) $$\begin{array}{c} \theta =\theta_{\text{old}}+\frac{0.25}{100}O(\theta_{\text{old}},Y)\\ s.t.\;0.25>\theta_{\text{high}}>\theta_{\text{low}}>0\\ O(\theta_{\text{old}},Y)=\left(\begin{array}{c} \Delta \theta_{\text{high}}\\ \Delta \theta_{\text{low}} \end{array}\right) \end{array}$$

(5) $$\begin{array}{c} \Delta \theta_{j}=\{\begin{array}{cc} 1 & \text{increase}\\ -1 & \text{decrease}\\ 0 & \text{no change} \end{array} \end{array}$$

(6) $$\begin{array}{c} \;j=\{\begin{array}{c} \text{high}\\ \text{low} \end{array} \end{array}$$

A manual operation was used when the assistant needed to confirm the prosthetic motion or hold the object securely.

### Control based on pattern recognition

**Figure 5:**
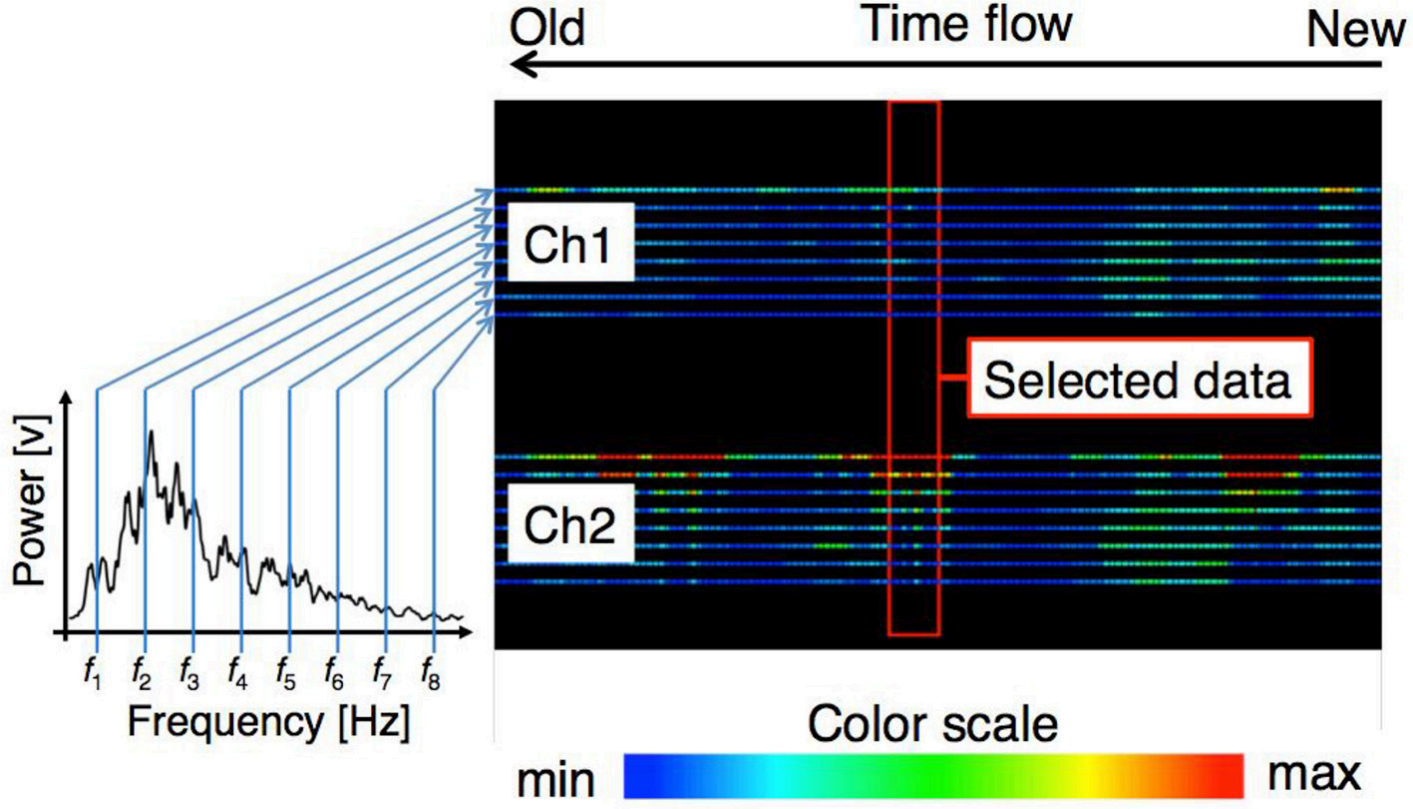
Graph of the pattern recognition control method. The right panel shows the time flow of feature levels of each sensor. Each line denotes the power of the electromyogram at a specific frequency. The color scale is shown at the bottom. The assistant selects the training data when the proper feature level appears within the central red box.

In the pattern recognition method, a 3-layer Artificial Neural Network (ANN) was determined by using the myoelectric potential frequency domain feature (Nishikawa et al., 1999). The feature quantity was transmitted to the computer of the external assistant and was drawn on the screen as a heat map, where the color varied with the power intensity (Figure 5). The assistant determined a feature vector that could be used as the training data and a hand motion label that was associated with the training data. The assistant used the heat map and the movement of the user to determine the appropriate training data. The prosthetic hand controller received the training data transmitted from the external terminal and learned using the received training data.

#### Frequency domain feature quantity of the myoelectric potential

The myoelectric feature value was calculated with a cycle of 10 ms, and recognition processing was performed using the latest feature level. In pattern recognition, the EMG measurements were performed with two sensors from the user forearm extensor group and flexor muscle group, and a second-order IIR high-pass filter with a cutoff frequency of 50 Hz was applied. A Fourier transformation was then carried out with a window width of 256 points, and power averages were obtained in the range of ±15.6 Hz centered on eight frequencies of 23.4, 46.9, 70.3, 93.8, 140.6, 187.5, 250.0, and 312.5 Hz (Jiang et al., 2014). Because eight values were obtained for each sensor, a 16-dimensional feature vector of 2 sensors × 8 dimensions could be obtained from one feature extraction. For the training data, 20 feature vectors for each hand motion were used.

#### Configuration of the ANN

The ANN consisted of three layers. The numbers of neurons in each layer were 16 for the input layer, 32 for the intermediate layer, and 8 for the output layer. To discriminate the hand motions, one of the neurons for the output layer was 0.95, and the other neurons were 0.05. At the time of recognition, when one of the output layer neurons reached the output value of 0.65 or more, the hand motion associated with the firing neuron was identified. A sigmoid function was used as the activation function of each neuron. An input signal was obtained by using the power of eight frequency bands of the frequency spectrum obtained by Fast Fourier Transform (FFT) of the myoelectricity for two sensors. An arbitrary hand movement was assigned as a label to 20 consecutive datasets, and it was used as training data. Every time training data was added, the ANN learned by the error backpropagation method together with existing training data. Iterative calculation was performed so that all training data was learned 200 times. The learning rate α was set to 0.1.

#### Graph drawing of the frequency domain feature quantity

The myoelectric characteristic level was transmitted to the PC at a cycle of 10 ms and plotted with the colors corresponding to the power intensity for each dimension of the feature vector. The data were transmitted 66 bytes at a time. The content of the transmission data was 1 byte for the start signal, a 16-dimensional feature vector of float type 64 byte, and 1 byte signaling termination character. After the PC received the data, the oldest buffered feature quantity was overwritten with the newest feature quantity. Then, the graph was updated. The plotted graph is shown in Figure 5. The values of each dimension were plotted in the vertical direction on the screen, and two bands composed of 8 × 2 lines represented the feature level of each channel. Of the eight lines, the power of the lower frequency band increased toward the top of the screen, and the power of the higher frequency band increased traveling down the screen. When a new value was measured, the graph flowed to the left. The graph at the right end of the screen represented the newest feature level.

#### Operation of the external terminal

The external assistant selected the feature quantity while looking at the terminal screen and determined the label of the hand movement associated with the operation. The determined label and training data were transmitted to the prosthesis controller and learned by the prosthesis controller. The training data used for learning were recorded on the external terminal with the label.

## Performance test and evaluation

Performance testing and evaluation of the discrimination rate were conducted for the threshold value and pattern discrimination control methods. In the test, the subjects were instructed to maintain a specific hand motion in a random order for 5 s. In addition, each hand motion was performed 10 times for each test. All experiments were approved by the ethics committee of The University of Electro-Communications (No. 10006) and The National Center for Child Health and Development (No. 756). In addition, written informed consent from parents of all child subjects was received.

### Performance test of the control method based on the threshold value

Three healthy 20-year-old males and one 5-year-old congenital left upper limb deficient boy were tested. The recognition operation was tested on two movements: grasping and opening. The sensors were aimed at the forearm flexor muscle group.

To verify the ability to adjust the threshold value accordingly, two types of experiments were performed. One was to adjust the threshold before each test, and the other used the same threshold after adjusting the threshold for the first test. The tests were performed six times for each method. The test set used an interval of 1 h. The first through third tests and the fourth through sixth tests were performed on different days.

In addition, the boy subject was tested once to collect reference data, and the experiments were carried out 5 times instead of 10 times for each manual operation to concentrate on the experiment. In addition, the boy subject had used the prosthesis of this system for approximately one year. To calculate the correct answer rate, an identification result of 4 s, excluding the first second, was used, taking into consideration the reaction time of the subject. In addition, when a feature level was in the interval between the threshold values and the insensitive area, the operation just before entering the insensitive area was outputted.

### Performance test of the control method based on pattern recognition

Three healthy 20-year-old male subjects performed the experiments. The identification operation was conducted once for the rest, grasping, and opening operations. The sensors were aimed at the forearm flexor muscle group and extensor group. The training data were selected from the myoelectric patterns when the subjects performed daily activities, such as holding daily necessities and playing. The state of motion of the subjects at the time of the myoelectric feature measurement and the type of muscles on which sensors were placed were used as the criteria for the selection of the training data (e.g., a grip motion if there was a response to the flexor group side sensor and an opening action if the extensor group side sensor responded). In addition, when the identified action was unidentifiable, it was deemed that the same action as the previously identified action was identified.

We verified whether the training data selected by the external assistant were training data that could be separated by the hand motion. A Principal Component Analysis (PCA) was performed on the training data selected by the same subject, and the mixing condition of the training data by the hand motion was confirmed.

### Application experiments

Application experiments on the threshold control of the 1-DOF myoelectric prosthetic hand system using PWAI with child upper limb amputee subjects were conducted. There were seven subjects, and the age at the start of the myoelectric prosthetic hand application and the amputation stump are listed in Table 1. Before use of the myoelectric prosthetic hand, an explanation of ~15 min was provided to the parents. After the explanation and while under the supervision of experts, application training of ~1 h was conducted with the parents and young children. Subsequently, the parents and young children had voluntary training at each household.

**Table 1:** Defect position of the child subjects and initial age of myoelectric prosthetics hand use.

**Subject**	**Age at first use**	**Amputate position**
1	1	Below left elbow
2	2	Below left elbow
3	3	Below left elbow
4	4	Below left elbow
5	4	Below right elbow
6	4	Right elbow
7	5	Below right elbow

## Result

### Experimental results of the control method based on the threshold

**Figure 6:**
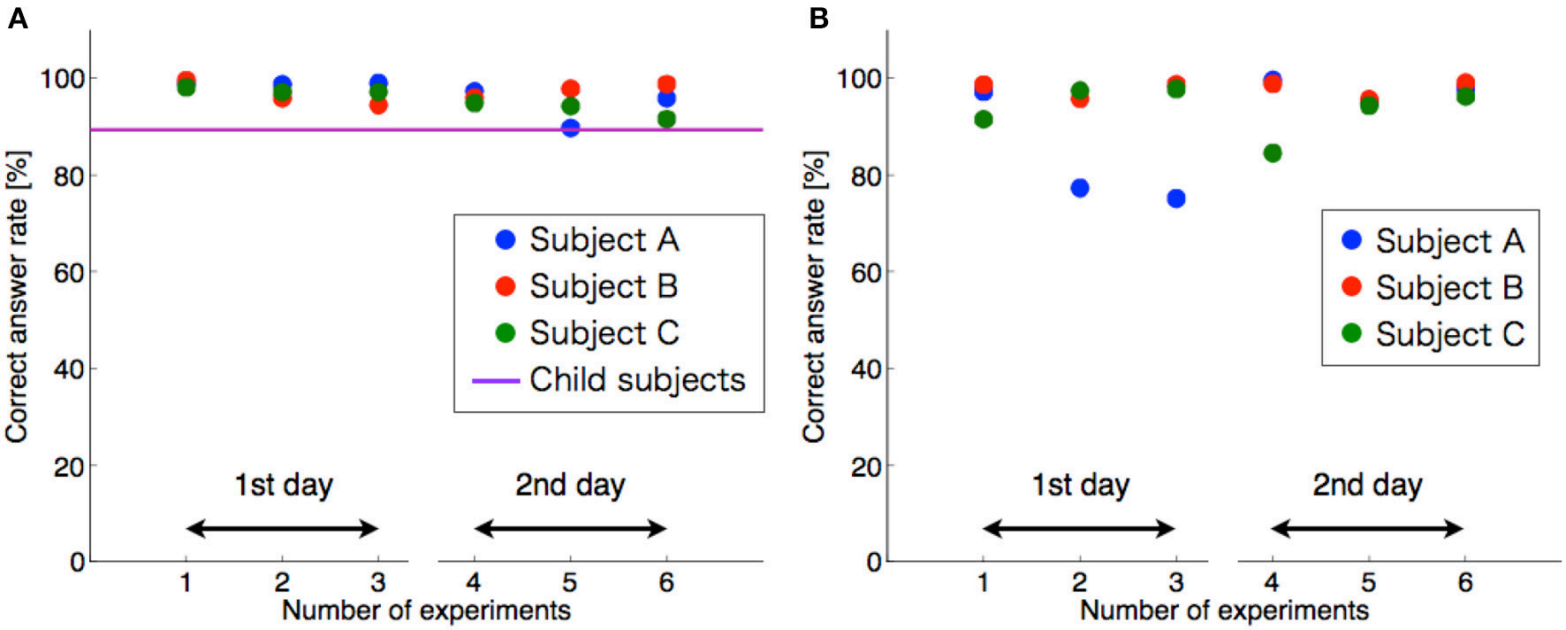
Discrimination rate of the threshold control method. **(A)** Shows the threshold adjustment for each test. **(B)** Shows the thresholds adjusted once. The horizontal and vertical axes denote number of experiments and correct answer rate, respectively. The circles are the data from each subject. The horizontal purple line indicates the results for child subjects.

The experimental results using the threshold control are shown in Figure 6. A high discrimination rate was recorded. The average discrimination rate was 94% when the threshold value was fixed, and the average discrimination rate was 96% when the threshold value was adjusted for each test. A statistical test with one-sample *t*-test supported the above results. The average discrimination rates of both conditions were significantly higher than chance level (50%) [adjusted each time, *p*-value: *p* = 9.83 × 10^−23^ <0.01, *t*-value: *t*_(17)_ = 73.6; adjusted once, *p* = 6.05 × 10^−15^ < 0.01, *t*_(17)_ = 25.3]. However, when the threshold was fixed, as shown in Figure 6B, a sharp decline in the discrimination rate (sub. A) occurred, and in an additional experiment, a drop in the discrimination rate occurred. For the experiment with the boy subject, the discrimination rate was 89%.

### Result of the performance test for the control method based on pattern recognition

**Figure 7:**
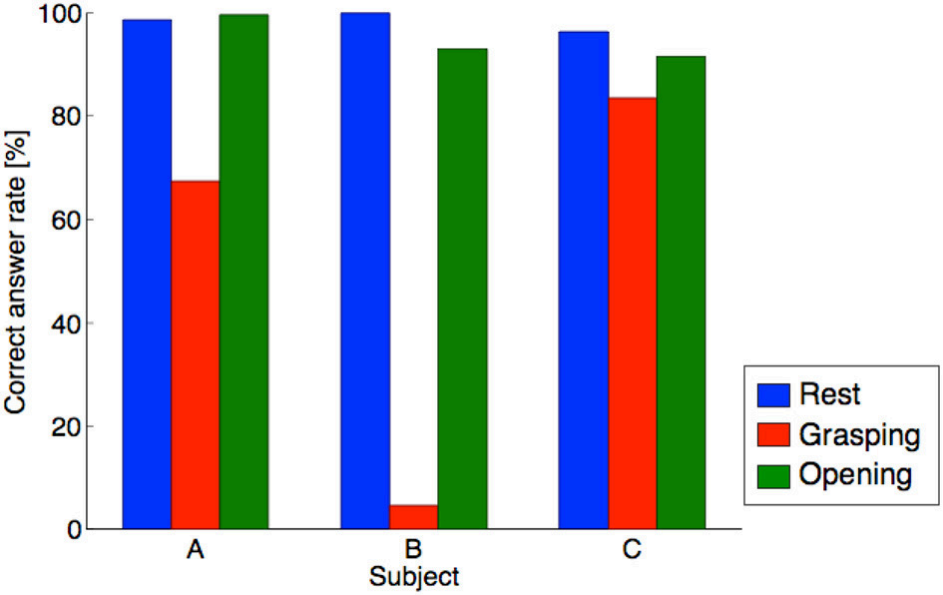
Discrimination rate of the pattern recognition method. The horizontal and vertical axes denote subject and correct answer rate, respectively. The blue, red, and green bar indicate correct answer rate in the resting, grasping, and opening conditions, respectively.

The results of the experiments using pattern recognition control are shown in Figure 7. The average discrimination rates in the three motion recognitions were 89, 66, and 90% for each subject, and the average discrimination rate was 82%. According to the *t*-test, the average discrimination rate was statistically higher than chance level (25%) [*p* = 5.47 × 10^−4^ < 0.01, *t*_(8)_ = 5.54]. The discrimination rate of the pattern control method was lower than that of the threshold method. The discrimination rate of subject B was low. The lower discrimination rate could be because of an inaccurate selection of the input data.

**Figure 8:**
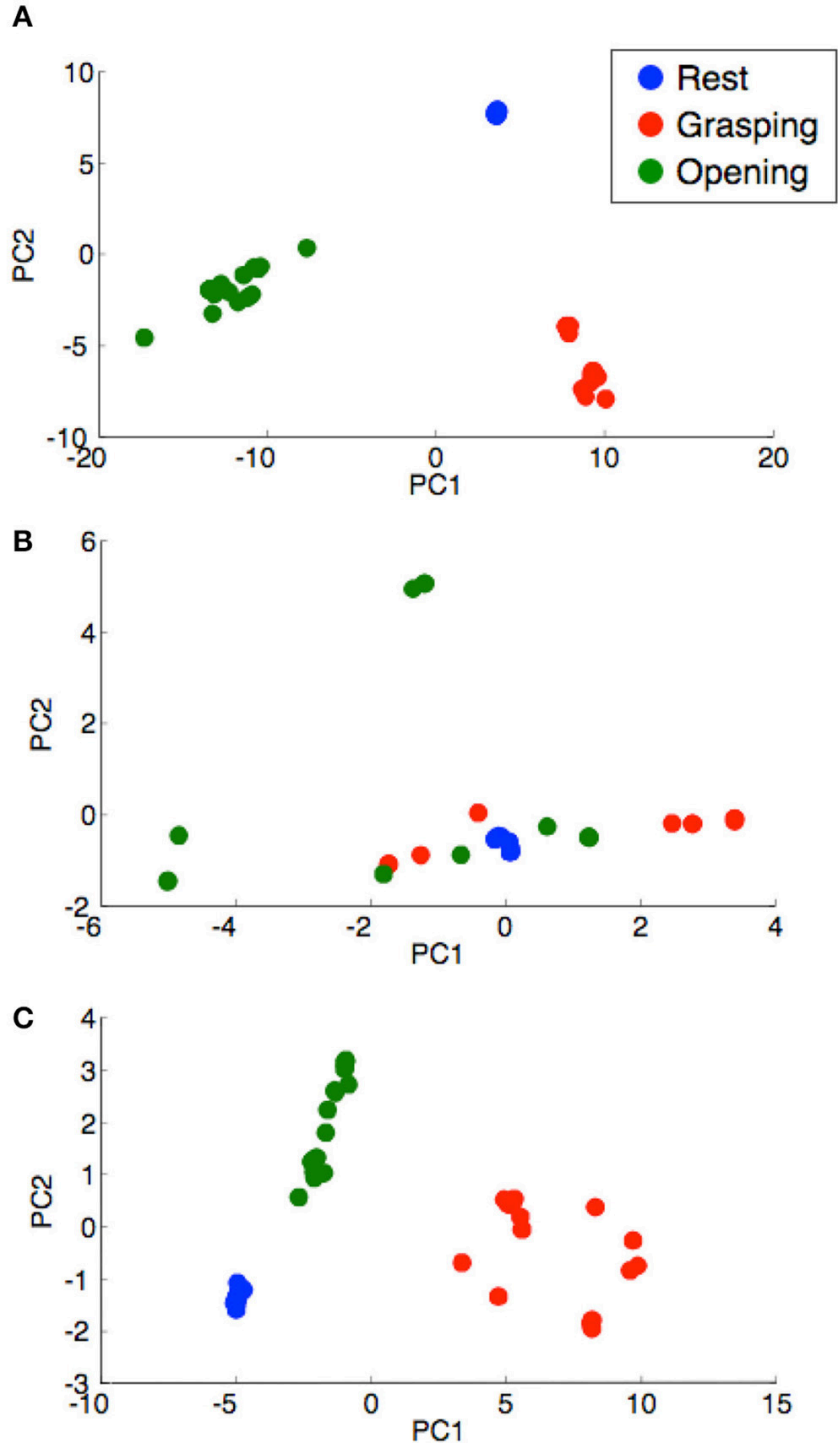
Distribution of the learning data for each subject. **(A)** Subject A, **(B)** subject B, and **(C)** subject C. The horizontal and vertical axes denote the principal components PC1 and PC2, respectively. The blue-, red-, and green-filled circles indicate PCs in the resting, grasping, and opening conditions, respectively.

Although the discrimination rate of grasping for subject B was abnormally low, the average discrimination rate exceeded 80%. To confirm the separation of the training data, a PCA was performed on the training data used for learning, and the figure with the horizontal axis as the first principal component and the vertical axis as the secondary principal component is shown in Figure 8. The cumulative contribution rates to the second principal component for each subject are listed in Table 2. The training data of subject B were difficult to separate by the hand motion. The training data of subject B were mixed in the feature space, and it was difficult to separate the training data.

**Table 2:** Cumulative contribution ratio of the feature vectors for each subject (Figure 8).

**Subject**	**Cumulative contribution ratio [%]**
A	93.0
B	69.7
C	94.0

### Result of application experiments

**Figure 9:**
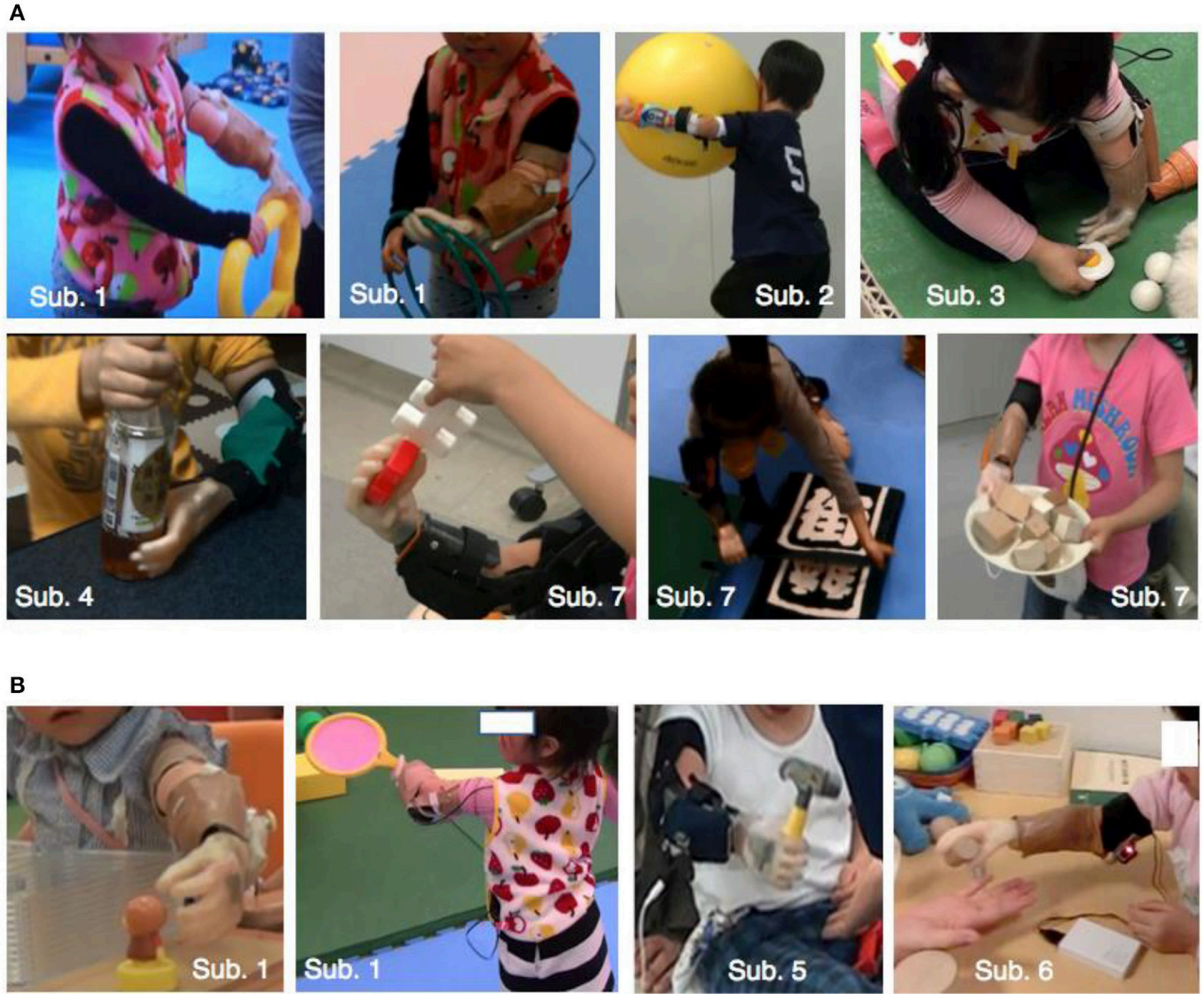
Children using the one-DOF prosthesis with the threshold control method. **(A)** Shows bimanual cooperative action. **(B)** Shows unimanual action. Subject's ID was shown in Table 1. Details of actions that subjects could perform are shown in Table 3.

Figure 9 shows the subjects using the myoelectric prosthetic hands with threshold control. According to Figure 9A, subjects could perform many kinds of bimanual daily movements, e.g., playing with a toy, holding a large object, towel folding, and opening a plastic bottle with the myoelectric prosthetic hand. Figure 9B also shows that subjects could also perform unimanual movements with myoelectric prosthetic hand, e.g., holding an object and reaching to grasp. In addition, we listed the behaviors that could be performed by a child subject using a myoelectric prosthetic hand in Table 3.

**Table 3:** Actions the subjects were able to perform.

Bimanual operation	Assistive motion	Zipper opening of the pouch Decomposition of block toys Tape cutting Cotton tearing Kitchen play Open a plastic bottle
	Object holding	Play with swing Play with push toys Hold a dish Hold paper
	Object manipulation	Embracing heavy objects (stuffed animals, balls, etc.) Ball throwing
Unimanual operation	Use of tools	Writing with a pen Shaking a hammer
	Object holding	Have accessories (dolls, confectionery, building blocks, etc.)
	Object manipulation	Removal of goods from boxes Transport of holding articles Building blocks play

## Discussion

### Control method based on the threshold

In the threshold control method, the system adequately controlled the prosthetic hands based on the high discrimination rate, including the boy subject. In addition, by adjusting for each discrimination test, and because the discrimination rate remained high, the proposed system could be appropriately adjusted by external assistants. The possible causes of the change in the discrimination rate when the threshold value was fixed could be a change in the sensor position, a change in the impedance of the sensor electrode, and the effect of sweat on the skin.

### Control method based on pattern recognition

For the motion classification of the hands with pattern recognition, the average discrimination rate was 82%, which was lower than the threshold control. In the pattern recognition method, because the number of recognition operations increased to three actions, including rest, the recognition difficulty level was increased. In addition, Figure 7 shows that the difference between the subjects was large. Based on the results of subject B in Figure 7, the discrimination rate of the grasping motion was low. This low discrimination rate could be caused by the selection error of the training data. The recognition of the grasping motion was difficult because the grasping motion training data selected was similar to other motions. From Figure 8 and Table 2, the training data used for discriminating the hand motion of subject B did not constitute a sufficiently separated electromyogram feature vector; however, the data were duplicated for each hand movement. It was difficult for an external assistant to choose features in the frequency domain using hand motion. A system that does not rely on assistant skill, such as judging feature vectors suitable for hand motion, is required.

## Conclusion

In this study, a myoelectric prosthetic hand control system that could be adjusted instantaneously by external assistants was proposed and evaluated to construct a usable myoelectric prosthetic hand system without requiring long-term training for child users. In the 1-DOF myoelectric prosthetic hand system that performed an opening or closing operation by a threshold control, the discrimination rate was 96% on average in adult subjects with appropriate adjustment of the threshold value. In addition, the young child subject recorded a high discrimination rate of 89%. The high discrimination rate was maintained by appropriately adjusting the discriminator based on the state of the user's myoelectric potential. In the hand motion recognition system, which enabled control of the multi-DOF by pattern recognition control, the discrimination rate of the three actions was 82%. Therefore, the selection of the training data was a difficult task for external assistants, and in future developments, automatic selection by a program and development of an auxiliary system that facilitates selection should be developed. With this system, the issues listed in the Introduction were solved as follows.

① The hand system must be usable by children who cannot understand verbal communications.

One-DOF myoelectric prosthetic hands, which contract/weaken the muscles, could be used by young children, ages 1–5 years (Figure 9).

② The hand system should contain functions of the direct control and pattern recognition methods.

In direct control, a system that monitors the myoelectric potential amplitude information and adjusts the threshold value was developed. In pattern recognition, a system that monitors the myoelectricity pattern in the frequency domain and selects the training data was constructed.

③ The parental switch should allow an external assistant to control the myoelectric prosthetic hand at any time.

A system that allowed assistants to select and execute the grasping, opening, and resting motions of the myoelectric prosthetic hand by the PWAI operation was constructed.

④ The hand system should be easy to use, and it should be usable by non-specialists (e.g., the parents of the users).

In the one-DOF myoelectric prosthetic hand system using threshold control, an application for an Android terminal was developed. Manual manipulation of the myoelectric prosthetic hands and adjustment of the threshold value was performed by operating the graphical user interface; therefore, parents could train the myoelectric prosthetic hands.

In pattern recognition, because the selection of the training data is difficult, a system for assisting the training data selection is required.

## Author contributions

YH designed the study, and wrote the initial draft of the manuscript. YM contributed to the development of children's hand prosthetic, and assisted in the preparation of the manuscript. All other authors have contributed to data collection and interpretation, and critically reviewed the manuscript. All authors approved the final version of the manuscript, and agree to be accountable for all aspects of the work in ensuring that questions related to the accuracy or integrity of any part of the work are appropriately investigated and resolved.

### Conflict of interest statement

The authors declare that the research was conducted in the absence of any commercial or financial relationships that could be construed as a potential conflict of interest.
